# Discovery of miRNAs and Development of Heat-Responsive miRNA-SSR Markers for Characterization of Wheat Germplasm for Terminal Heat Tolerance Breeding

**DOI:** 10.3389/fgene.2021.699420

**Published:** 2021-07-28

**Authors:** Pooja Sihag, Vijeta Sagwal, Anuj Kumar, Priyanka Balyan, Reyazul Rouf Mir, Om Parkash Dhankher, Upendra Kumar

**Affiliations:** ^1^Department of Molecular Biology, Biotechnology and Bioinformatics, College of Basic Sciences and Humanities, CCS Haryana Agricultural University, Hisar, India; ^2^Centre for Agricultural Bioinformatics, ICAR-Indian Agricultural Statistics Research Institute, New Delhi, India; ^3^Department of Botany, Deva Nagri College, Meerut, India; ^4^Division of Genetics and Plant Breeding, Sher-e-Kashmir University of Agricultural Sciences and Technology, Srinagar, India; ^5^Stockbridge School of Agriculture, University of Massachusetts, Amherst, MA, United States

**Keywords:** *Triticum aestivum* L., genetic diversity, simple sequence repeats, population structure, marker assisted breeding, heat responsive genes

## Abstract

A large proportion of the Asian population fulfills their energy requirements from wheat (*Triticum aestivum* L.). Wheat quality and yield are critically affected by the terminal heat stress across the globe. It affects approximately 40% of the wheat-cultivating regions of the world. Therefore, there is a critical need to develop improved terminal heat-tolerant wheat varieties. Marker-assisted breeding with genic simple sequence repeats (SSR) markers have been used for developing terminal heat-tolerant wheat varieties; however, only few studies involved the use of microRNA (miRNA)-based SSR markers (miRNA-SSRs) in wheat, which were found as key players in various abiotic stresses. In the present study, we identified 104 heat-stress-responsive miRNAs reported in various crops. Out of these, 70 miRNA-SSR markers have been validated on a set of 20 terminal heat-tolerant and heat-susceptible wheat genotypes. Among these, only 19 miRNA-SSR markers were found to be polymorphic, which were further used to study the genetic diversity and population structure. The polymorphic miRNA-SSRs amplified 61 SSR loci with an average of 2.9 alleles per locus. The polymorphic information content (PIC) value of polymorphic miRNA-SSRs ranged from 0.10 to 0.87 with a mean value of 0.48. The dendrogram constructed using unweighted neighbor-joining method and population structure analysis clustered these 20 wheat genotypes into 3 clusters. The target genes of these miRNAs are involved either directly or indirectly in providing tolerance to heat stress. Furthermore, two polymorphic markers miR159c and miR165b were declared as very promising diagnostic markers, since these markers showed specific alleles and discriminated terminal heat-tolerant genotypes from the susceptible genotypes. Thus, these identified miRNA-SSR markers will prove useful in the characterization of wheat germplasm through the study of genetic diversity and population structural analysis and in wheat molecular breeding programs aimed at terminal heat tolerance of wheat varieties.

## Introduction

*Triticum aestivum* L. is the most commonly used hexaploid bread wheat (AABBDD; 6X = 2*n* = 42) with genome size of approximately 17 GB. It is derived via crossing among tetraploid *Triticum turgidum* (AABB) and diploid *Aegilops tauschii* (DD), whereas *T. turgidum* (AABB) was derived via crossing between *Triticum urartu* (AA) and *Aegilops speltoides* (BB) (Petersen et al., 2006). Wheat is the second principal staple food cereal of the world fulfilling the maximal carbohydrates dietary requirement (Kumar et al., 2013). To meet the food demand of the rapidly growing population, wheat productivity should be enhanced by 40–50% by 2050 (Sharma et al., 2021). Terminal heat stress is the serious issue in wheat-cultivating regions due to the rise in temperature every year because of the global warming effect (Mitra and Bhatia, 2008). Heat stress at the time of anthesis and grain filling adversely affects wheat quality and yield in terms of grain number and grain weight (Ferris et al., 1998; Kumar et al., 2013). A reduction of 4–6% in wheat grain yield was observed for every 1°C increase in temperature (Zhao et al., 2017). High temperature at the time of flowering reduces its fertility and disrupts photosynthesis mainly through leaf senescence, leading to heavy yield losses (Fábián et al., 2019). Substantial variation for heat tolerance is available among wheat cultivars and breeding lines but remains relatively unexploited. In comparison to the heat-susceptible lines, the genotypes maintaining yields under higher temperatures must contain genes that prevent degradation of chlorophyll molecules, maintain low canopy temperature, and encode enzymes that can maintain activity under elevated temperatures.

The heat tolerance capability of a genotype depends on different physiological, biochemical, and genetic parameters, which are directly or indirectly involved in providing defense against heat stress (Kumar R. R. et al., 2017). The microRNAs, transcription factors (TFs), signaling molecules, and heat-shock proteins (HSPs) play key roles in providing thermotolerance (Kumar et al., 2012). The microRNAs are small (19–24 bp), non-coding RNA molecules synthesized endogenously from *microRNA* (*miRNA*) genes and present in both prokaryotes and eukaryotes (Rhoades et al., 2002; Tyagi et al., 2019). It is well known that the miRNAs control the gene expression either by direct cleavage of the target mRNA or its suppression at translational level (Bej and Basak, 2014). These are vital regulatory elements intricate in several biotic and abiotic stress responses (Singh and Shah, 2014; Jatan and Lata, 2019). Numerous abiotic-stress-associated miRNAs have been recognized and characterized in plants, whereas only few reports are available in wheat (Ohama et al., 2017). In the case of abiotic stress, a strong interaction between miRNAs and TFs has been noticed. The miRNAs and TFs interaction regulate the expression of a particular gene either by its upregulation or downregulation in a significant manner (Chow et al., 2016). Earlier studies have identified miRNAs in response to different heat stress treatment at the grain filling stage of several heat-affected and heat-tolerant wheat varieties (Qin et al., 2008; Xin et al., 2010; Kumar et al., 2015; Ragupathy et al., 2016).

Previous studies have also shown the association of simple sequence repeats (SSRs) with heat-stress-responsive traits (Hassan, 2016; Sharma et al., 2018; Al-Ashkar et al., 2020; Saha et al., 2020). SSRs, also called as microsatellites are one- to six-nucleotide long tandem repeats distributed randomly throughout the genome (Gupta et al., 1996; Chen et al., 2009). SSRs have been identified in the non-coding, coding, and untranslated regions (UTRs), but their abundance was reported highest in non-coding regions of the genomes (Madsen et al., 2008). SSRs are widely used as molecular markers for genetic diversity analysis, marker-assisted selection, and linkage mapping among populations (Mir et al., 2012a, b, 2013; Gupta et al., 2013; Mir and Varshney, 2013; Kumar et al., 2021). The following properties of SSRs make them the best marker of interest in biological researches: (1) codominant nature (able to distinguish between homozygous and heterozygous); (2) divergence in number of short tandem repeats; (3) presence of multiple alleles; (4) polymorphic nature; and (5) high reproducibility (Brandström et al., 2008; Heesacker et al., 2008; Mir and Varshney, 2013; Mir et al., 2013). Genic SSR markers designed from heat-stress-associated genes were screened over heat-tolerant and heat-susceptible wheat genotypes to study the genetic diversity and association between SSR markers and traits of heat stress tolerance (Sharma et al., 2020; Manjunatha et al., 2021). Previous reports confirmed the presence of SSRs in pre-miRNA sequences of many plant species including *Arabidopsis*, rice, wheat, and *Brassica* in response to different abiotic stresses (Ganie and Mondal, 2015; Kumar A. et al., 2017; Singh et al., 2017; Tabkhkar et al., 2020). There are very few reports for genetic diversity studies in heat-tolerant and heat-responsive wheat genotypes that used miRNA-SSR as molecular markers. Tyagi et al. (2021) mined 96 heat-responsive miRNAs that led to the development of 13 miRNA-SSR markers. These markers were used to study the genetic diversity, population structure, and characterization of 37 wheat genotypes for heat tolerance breeding. Sharma et al. (2021) also designed 177 SSR markers from heat-responsive genes and pre-miRNAs (11) of wheat genome and screened over 36 wheat genotypes to study genetic diversity for heat tolerance (Sharma et al., 2021). The results of the previous studies suggested the potential role of miRNA-derived SSR markers in marker-assisted breeding (MAB), which aimed to improve heat tolerance and adaptability developmental traits in crop plants including wheat (Sharma et al., 2021; Tyagi et al., 2021).

Keeping this in view, the present study was planned to develop more miRNA-derived SSR markers for the identification of additional promising markers for wheat molecular breeding programs. We mined heat-responsive miRNA from different plant species, including *Arabidopsis*, rice, and wheat, and developed miRNA-SSRs markers from the flanking region of pre-miRNA of wheat genome. The genetic diversity of 20 genotypes of wheat that differs in temperature response was evaluated using 70 miRNA-SSR markers. We have also studied the target genes of heat-responsive miRNAs and their role in heat stress. Our study will help in the categorization of the terminal heat-tolerant and heat-susceptible genotypes using miRNA-SSRs markers in the early stages of wheat development.

## Materials and Methods

### Plant Material and DNA Extraction

Twenty wheat genotypes comprising 13 terminal heat-tolerant and 7 heat-susceptible genotypes were used in this study (Table 1). One hundred milligrams of fresh leaves was used for DNA isolation using cetyl trimethylammonium bromide (CTAB) method (Doyle and Doyle, 1987). The samples were immersed in liquid nitrogen and ground to fine powder using a Tissue Lyser (Retsch Mixer Mill MM400) machine. CTAB buffer (containing 2% CTAB, 1.4 M NaCl; pH 8, 20 mM EDTA, and 100 mM Tris Cl) was added to the ground powder and incubated at 65°C for 1 h. Chloroform/isoamyl alcohol (24:1) was mixed properly with lysed sample and centrifuged at 7,800 rpm for 10 min. The supernatant was collected in a fresh tube and added prechilled iso-propanol to precipitate the genomic DNA. After centrifugation, pellet was washed two times with 70% alcohol. The DNA pellet was dried and dissolved in 100 μl Tris–EDTA (TE) buffer. DNA quality and quantity were checked using NanoDrop spectrophotometer (Thermo Scientific, United States) along with 0.8% agarose gel separation. To perform polymerase chain reaction, DNA was diluted with nuclease-free water (NFW) to 100 ng/μl concentration.

**TABLE 1:** Details of 20 terminal heat-tolerant and heat-susceptible wheat genotypes used in this study.

**S. no.**	**Genotypes**	**Tolerant/susceptible**	**Pedigree**	**Developing center**
1	Cv. Raj3765	Tolerant	HD2402/VL639	RAU, Durgapur
2	WH730	Tolerant	CPAN2092/IMPROVED LOK1	CCSHAU, Hisar
3	WH1021	Tolerant	GW296/SONAK	CCSHAU, Hisar
4	WH1218	Tolerant	KA/NAC//TRCH/3/VORB	CCSHAU, Hisar
5	PBW533	Tolerant	PBW343/PBW138//PBW343	PAU, Ludhiana
6	WH789	Tolerant	–	CCSHAU, Hisar
7	WH1142	Tolerant	CHEN/Ae.Sq. (TAUS)//FCT/3/2*WEAVER	CCSHAU, Hisar
8	WH1124	Tolerant	MUNIA/CHTO/AMSEL	CCSHAU, Hisar
9	WH1133	Tolerant	BABAX/LR42//BABAX*2/3/VJV1TS1	CCSHAU, Hisar
10	WH787	Tolerant	–	CCSHAU, Hisar
11	WH147	Susceptible	E4870-C303/S339-PV18	CCSHAU, Hisar
12	WH711	Susceptible	ALD “S”’HUAC//HD2285/3/HFW-17	CCSHAU, Hisar
13	HD2967	Tolerant	ALD/COC//URES/HD2160M/HD2278	IARI, New Delhi
14	WH157	Susceptible	NP876/S308//CNO/8156	CCSHAU, Hisar
15	WH542	Susceptible	JUP/BJY “S”//URES	CCSHAU, Hisar
16	WH1140	Tolerant	WBLLI*2/VIVITSI	CCSHAU, Hisar
17	PBW550	Tolerant	WH594/RAJ3858/W485	PAU, Ludhiana
18	WH283	Susceptible	HD-1981/RAJ-821	CCSHAU, Hisar
19	HD2687	Susceptible	Gene introgressed from Sr31 Lr26 Yr9 Pm8	IARI, New Delhi
20	HD2329	Susceptible	HD1962/E4870/3/K65/5/HD1553/4/UP262	IARI, New Delhi

### PCR Amplification

To study the polymorphism, DNA of all 20 wheat genotypes was amplified using an Eppendorf thermal cycler. A reaction mixture of 20 μl containing 10 μl 2× Promega green master mix, 2 μl template DNA (200 ng), 0.4 μl of each forward and reverse primer (0.2 μM), and 7.2 μl NFW was prepared. PCR conditions were as follows: initial denaturation at 94°C for 3 min, 35 cycles of denaturation at 94°C for 50 s, annealing at 50–56°C (differ with primer pair) for 30 s, and extension at 72°C for 40 s, followed by the final extension at 72°C for 7 min. Amplified PCR product was resolved on 10% denaturing urea polyacrylamide gel electrophoresis unit (urea PAGE) at 120 V for 4 h with 1× Tris–acetate–EDTA (TAE) buffer. After staining with ethidium bromide (EtBr), the gel was visualized in Vilber Fusion Solo S gel documentation system.

### Screening of Heat-Responsive miRNAs and Designing of miRNAs-SSRs Markers

**FIGURE 1:**
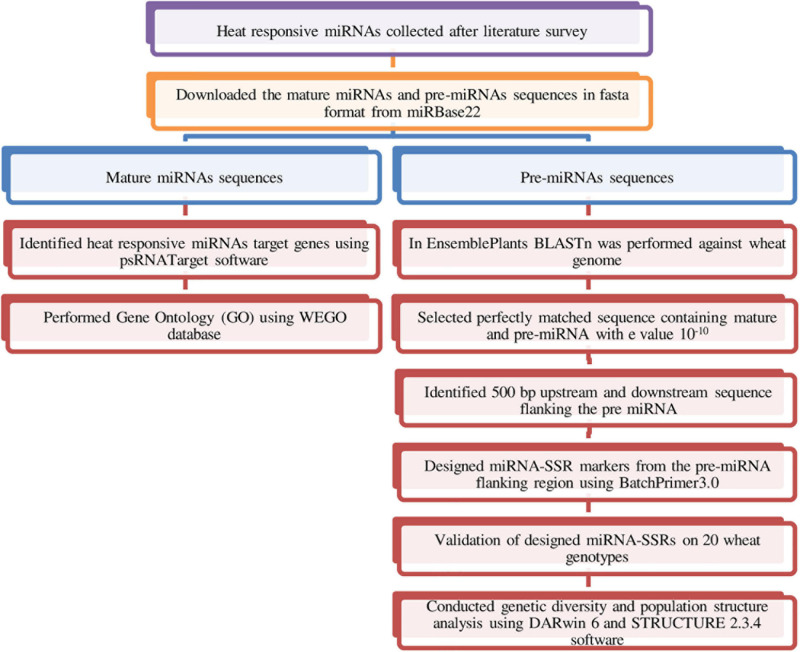
Pipeline describing the process of identification and validation of miRNA-SSRs in wheat.

Heat-responsive miRNAs reported in different plants including *Arabidopsis*, wheat, rice, maize, and *Brassica* were collected based on extensive literature survey. Mature and stem loop (pre-miRNA) sequences of these heat-responsive miRNAs were obtained from miRBase v22 database^1^ in FASTA format (Kozomara et al., 2019). In Ensembl Plants database,^2^ BLASTn search was carried out against reference genome data of wheat using pre-miRNA sequences to get the 500-bp downstream and 500-bp upstream sequence flanking pre-miRNA (Bolser et al., 2015). BLASTn search resulted into multiple hits; among them, the best sequence that aligned perfectly (100%) to the wheat genome and has an *e*-value of 10^––10^ was selected for designing of SSR markers. The microRNA-SSR markers were designed from the pre-miRNA flanking sequence using BatchPrimer3 v1.0.^3^ For primer synthesis di-, tri-, tetra-, penta-, and hexa-SSRs of minimum 12 bp length were chosen. The following parameters were set for designing of SSR flanking primers: primer length of 18–25 bp, melting temperature (T_m_) in 50–65°C range, 40–60% GC content, and product size of 100–300 bp length (You et al., 2008). A summary of the methods used for miRNA-SSR development and validation in wheat is represented in Figure 1.

### Allele Scoring and Genetic Diversity Analysis

A total of 70 miRNA-SSR markers were screened on genomic DNA of selected 20 wheat genotypes to study the polymorphism pattern in terminal heat-tolerant and heat-susceptible wheat genotypes. The scoring of all the polymorphic primers was done based on the absence and presence of DNA band. The absence and presence of DNA band was indicated as 0 and 1, respectively. The size (bp) of each DNA band was determined using Evolution Edge software based on the size of Quick-Load^®^ Purple 50 bp DNA Ladder bands. A binary (0/1) allele scoring matrix generated from polymorphic miRNA-SSR markers was used to compute the polymorphic information content (PIC) and genetic diversity among 20 wheat genotypes. PIC was calculated using the GeneCalc online bioinformatics tool^4^ (Nagy et al., 2012) according to the equation: *PIC* = 1−∑*pi*^2^, where *pi* is the frequency of *i*th allele of a particular locus. The phylogenetic relations between terminal heat-tolerant and heat-susceptible genotypes were drawn using DARwin v6.0 software (Perrier et al., 2003). Dissimilarity matrix was computed and used for the grouping of 20 genotypes or for the construction of dendrogram using unweighted neighbor joining followed by bootstrapping with 1,000 replications (Gascuel, 1997). Principal coordinate analysis (PCA) was also computed to categorize these 20 genotypes into different groups using DARwin v6.0.

### Population Structure Analysis

The total number of subpopulations present in a population of 20 wheat genotypes was identified using the Bayesian method with binary data matrix of 19 polymorphic miRNA-SSRs. This analysis was done using STRUCTURE 2.3.4 software (Pritchard et al., 2010). The following parameters were set: (1) burnin iteration length of 100,000, (2) Markov chain Monte Carlo (MCMC) value of 100,000, and (3) the value of subpopulations number (*k*) ranged from 1 to 10 with 3 repetitions (Chen et al., 2012). The best fit value of subpopulations was derived with the Evanno method (Evanno et al., 2005) using online STRUCTURE HARVESTER software^5^ (Earl and Bridgett, 2012). Genotypes with more than 80% similarity were allocated in a distinct subpopulation, and those with <80% were designated as admixture.

### miRNA Target Identification and Gene Ontology

The target genes of heat-responsive miRNAs were predicted using the psRNATarget server^6^ (Dai et al., 2018). Mature miRNA sequences in fasta format extracted from miRBase v22 were used as a query to find the targets in the wheat cDNA library. The following default parameters were used in search of target genes: (1) expectation value was set 5; (2) range of translational inhibition as 10--11 nucleotides; (3) G:U pair penalty, 0.5; (4) seed region at 2--13 nucleotides and maximum two mismatches were allowed in the seed region; (5) complementarity scoring length (HSP size), 19; and (6) energy to unpair (UPE) the target sequence, 25. Identified targets of heat-responsive miRNAs were used for Gene Ontology (GO) studies with Web Gene Ontology Annotation Plot (WEGO 2.0^7^) database (Ye et al., 2018). The Gene Ontology ID of the target genes for biological process, molecular function, and cellular component were retrieved from the Ensemble plant database and used for GO analysis in WEGO 2.0 using default parameters. A summary of the methods used for miRNA target identification is represented in Figure 1.

### Trait Data Recording and Analysis

The set of 20 genotypes including 13 heat-tolerant and 7 heat-susceptible genotypes were also phenotyped for 5 most important physiological/biochemical traits related to heat tolerance. The data on these traits were recorded under normal and late sown conditions at 10 and 20 days after anthesis. The canopy temperature depression (CTD) and chlorophyll content was recorded using an infrared thermometer and SPAD-502, respectively, whereas relative water content (RWC), electrolyte leakage to test cell membrane stability, and proline content were measured using established methods (Barr and Weatherley, 1962; Bates et al., 1973; Dionisio-Sese and Tobita, 1998).

## Results

### Development of Heat-Responsive miRNA-SSRs

**FIGURE 2:**
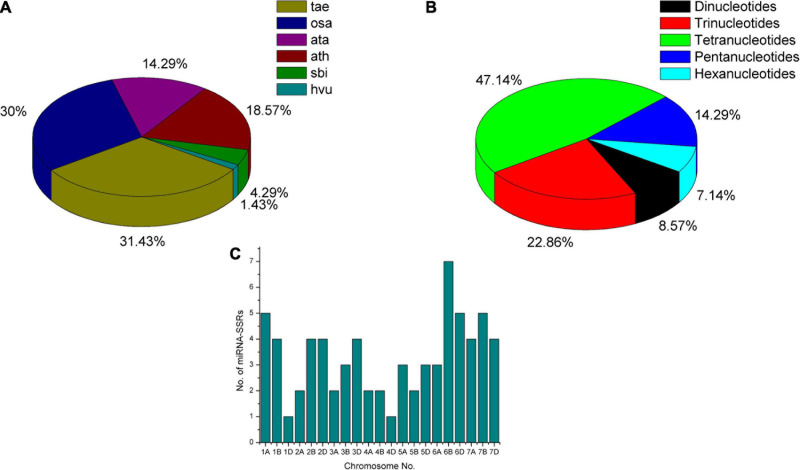
Frequency and distribution of heat-responsive miRNA genes with SSR motifs. **(A)** Frequency of heat-responsive miRNAs submitted in miRBase reported in various crops. **(B)** Frequency of wheat miRNA-SSR repeats present in miRNA genes. **(C)** Distribution of heat-responsive miRNA-SSRs on wheat.

From the literature survey, 80 heat-responsive miRNA families consisting of 104 members were recognized in *Arabidopsis*, wheat, rice, maize, and sorghum crops (Supplementary Table 1). Due to the lack of research on wheat miRNAs, very few wheat miRNA sequences were deposited in the miRBase v22 database. Therefore, along with *T. aestivum*, we also preferred the mature miRNAs and pre-miRNAs sequences deposited for *Oryza sativa (osa)*, *A. tauschii (ata)*, *Arabidopsis thaliana (ath)*, *Sorghum bicolor (sbi)*, and *Hordeum vulgare (hvu)* with frequency shown in Figure 2A. Mature sequences of five miRNAs (miR6941, miR3182, miR2012, miR2020, and miR2006) were not found in the miRBase v22 database. Primer designing using BatchPrimer3 v1.0 resulted in 70 significant miRNA-SSR primers pair, whereas no SSR flanking primers were obtained for two miRNAs (miR5071 and miR1122), and the absence of SSRs were noticed in 27 pre-miRNA sequences. The number of SSRs varies from dinucleotides to hexanucleotides with maximum dinucleotides frequency, i.e., up to 27 times (NN)_27_ and tetranucleotides repeated up to 10 times (NNNN)_10_. The maximum number of *miRNA* genes was found to possess tetranucleotides (47%) followed by trinucleotides (23%), pentanucleotides (14%), dinucleotides (9%), and hexanucleotides (7%) SSRs (Figure 2B). The highest number of miRNA-SSR markers was found to be located on 6B wheat chromosome and lowest on chromosome numbers 1D and 4D as shown in Figure 2C.

### Validation of miRNA-SSR Markers and Genetic Diversity Analysis

**FIGURE 3:**
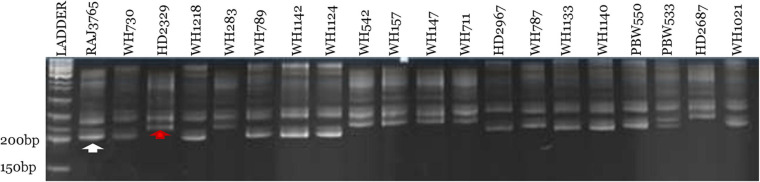
Denaturing 10% urea-PAGE profile represents polymorphic pattern of miR159c miRNA-SSR marker over 20 terminal heat-tolerant/heat-susceptible wheat genotypes. First lane represents 50 bp DNA ladder, and the white and red arrow denote terminal heat-tolerant and heat-susceptible wheat genotypes, respectively.

**FIGURE 4:**
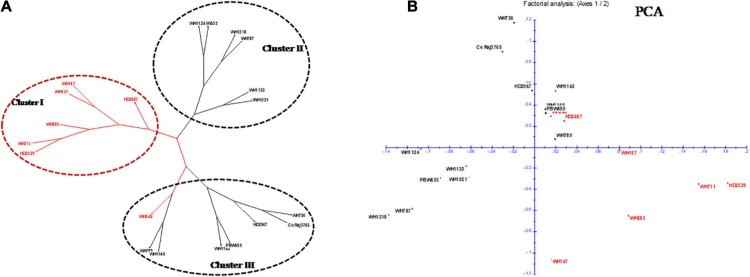
Phylogenetic studies of wheat genotypes with heat-responsive miRNA-SSRs. **(A)** Dendrogram showing clustering of 20 wheat genotypes using polymorphic miRNA-SSR markers. Red color indicates heat-susceptible wheat genotypes, and black color indicates heat-tolerant wheat genotypes. **(B)** 2-D plot of principal coordinate analysis (PCA) for all 20 wheat genotypes. Red and black colors were used for heat-susceptible and heat-tolerant genotypes, respectively.

Out of 70 screened miRNA-SSR markers, 64 markers resulted into clear, bright, and reproducible bands in all the 20 wheat genotypes (Supplementary Table 2). Among these, 19 polymorphic miRNA-SSR primers were chosen and used for genetic diversity studies of 20 terminal heat-tolerant and heat-susceptible genotypes (Table 2). Two polymorphic markers, miR159c and miR165b, were found to be able to distinguish terminal heat-tolerant and heat-susceptible wheat genotypes by the presence of different alleles in them (Figure 3). A total of 61 SSR loci were identified using 19 polymorphic primers pair with the range of 2–9 alleles, averaged 2.9 alleles per locus, and the amplicon size of polymorphic alleles ranging from 130 to 470 bp. The PIC value ranged from 0.10 to 0.87, with miR830 having the highest PIC (0.87) and miR159b and miR824 and miR2122 possessing the lowest PIC (0.10). The binary scoring matrix data obtained from 19 polymorphic miRNA-SSRs was used for phylogenetic studies of 20 terminal heat-tolerant and heat-susceptible wheat genotypes using DARwin6 software. The results clustered these 20 genotypes into 3 major clusters represented as clusters I, II, and III. Clusters I and II were found to consist of six terminal heat-susceptible and heat-tolerant wheat genotypes, respectively, whereas cluster III comprises of seven terminal heat-tolerant and one terminal heat-susceptible wheat genotype (Figure 4A). The principal coordinate analysis conducted using DARwin6 also distinctly separated the 20 wheat genotypes from each other (Figure 4B).

**TABLE 2:** Details of 19 polymorphic miRNA-SSR markers used in the study of 20 terminal heat tolerant/susceptible wheat genotypes.

**miRNA-SSR primer**	**SSR motif repeat**	**No. of alleles**	**Forward primer sequence (5′–3′)**	**Reverse primer sequence (5′–3′)**	**T_m_ (°C)**	**Amplicon size (bp)**	**PIC value**
miR159	(TTCT)4	4	CTCACCCCTCTATAAAACGAC	CTACATCTATGGGGCTAGGAG	50	140–260	0.62
miR159b	(CTC)4	2	ATTTTCCTTTCAATGACACCT	AAGAGATGGAACGGAAACTAC	51	165–169	0.10
miR159c	(CT)27	3	CTTTCCCTCGTGCTTGGAT	GCATAGTGATTTGATTTTCTTGTTAGC	52	180–250	0.61
miR159f	(CT)27	3	ACCTGTATAGGTTTTGCATGA	TTAGGTGCAGACTGAAAACAT	51.5	130–162	0.62
miR164a	(GCCC)3	7	ACTGCACTGCACGTGTTCTT	TTGAAGACGCATACCTCGTG	59.5	135–315	0.81
miR165b	(ATAC)10	6	CAACGGTGTGATTGTAAAAA	CGAAGTTTAATTTGGTTATGC	50	146–280	0.72
mir171b	(ATTG)3	3	CTGAACGCTACTGAGCCACT	CTACCAACACGGCAGCACTA	55	178–300	0.65
mir172c	(TTC)5	3	CCTCTCTTTGTCTTCATCCA	AAGAACCGACTGTGATCTGA	51.5	130–180	0.25
mir393a	(GAGG)4	2	CCTATATAAGGACCTCACTGGA	AGGCATTGTTGCTCTCTCT	52	157–175	0.49
mir396d	(GTGC)3	6	AAGTTATATCGGACCGTGTG	AGGAAGGGGTCGTATAAATAG	52	150–470	0.79
mir404	(TGCCGC)3	2	CTAAACCGGATAAAGGGTAGA	CAGAGGAACGCACGTAGT	52	140–162	0.49
mir830	(AGGGA)3	9	AGTACGCCTTGATCTCCTCT	CTACGTTACCTTCCTCTCTCC	53.5	130–320	0.87
mir824	(TTAA)3	2	GGCCTTTGACTGAATTAGTGT	CCAGAAAGGAATTATTTTGGA	50	134–140	0.10
miR857	(TTTA)3	2	TTCTTGCCTACTTGTTTCTG	GTCGCCGTCTTTGAATTT	50	267–272	0.10
mir1130a	(TGCA)3	2	AGTTGCACTGCTACAAGCTAC	ACTTTCGGATGTACTGTTCTG	52	146–165	0.48
mir2102	(GCC)4	2	ACCGCTGCTGTTGTATTG	TCAAGTCCTCTGCAAACAC	52	167–175	0.48
mir2122	(TTCTT)3	2	GGGTGGACAGTAAAATCAGA	CCATTTTTCAGGATCATTCTT	50	157–162	0.10
mir5384	(CCG)4	3	AGGGGATCCTCCTCAGAT	AGGTGGTTGGTGGCCAAG	52	280–350	0.66
mir9662b	(CGC)5	2	CCTTCACCAAACCCTCTT	GAGATCCAGCAGAAGGAGAT	53	227–235	0.18

### Population Structure Analysis

**FIGURE 5:**
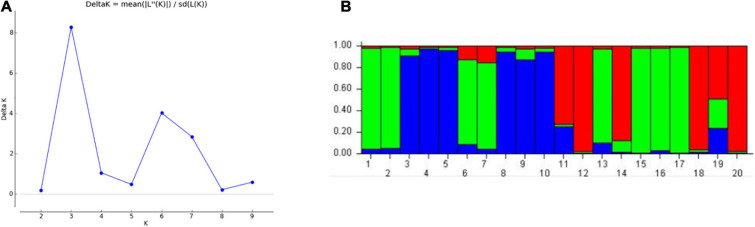
Evaluation of number of subgroups based on “STRUCTURE” output. **(A)** Delta *K*, best value of *K* = 3. **(B)** Bar plot showing distribution of 20 wheat genotypes into 3 subgroups as shown in green, blue, and red colors.

The population structure of 20 genotypes was studied using “STRUCTURE” software Bayesian clustering approach. The binary scoring matrix data of 19 polymorphic miRNA-SSRs screened on 20 genotypes was analyzed using this software, and the best “*K* = 3” value was selected based on Δ*K* value obtained from “Structure Harvester” (Figure 5A). This analysis distributed the population of 20 wheat genotypes into 3 subpopulations (P1, P2, and P3). Among these three subpopulations, each of P1 and P2 consists of six genotypes, and P3 contains four genotypes, whereas four genotypes were found to be admixed (Figure 5B). These results were obtained in accordance to the cluster analysis and PCA results.

### Identification of miRNA Target Genes

The target genes of heat-responsive miRNAs were identified using psRNATarget server (Supplementary Table 3). The 80 target genes identified using this server will play a crucial role in providing tolerance against heat stress at various stages of wheat growth, whereas most of the targets were related to grain filling stages. These miRNAs regulate the target genes either by its cleavage or by suppressing the translational mechanism. The four miRNAs, namely, miR1130a, miR528, miR1137a, and miR159a, targeted the heat shock protein 17 (hsp17), heat shock protein 90 (hsp90), heat stress associated 32-kD protein (hsa32), and DnaJ heat shock family protein, respectively. Many heat-responsive genes were found to have the common target of more than two miRNAs such as peroxidase, glycosyltransferase, phytochrome, phenylalanine, flavin containing monooxygenase, and sucrose synthase. The miRNAs were also found to regulate the signaling molecules including the mitogen-activated protein kinase (MAPK), zinc finger protein (ZFP), cyclin-dependent kinase inhibitor (CDKI), serine/threonine-protein kinase, CASP-like protein, and TFs such as Auxin response factor (ARF), WRKY, NAC 6A, ethylene responsive factor 5a (ERF 5A), and R1R2R3-MYB. Heat stress induces oxidative damage, and plants adapt to this by synthesizing antioxidants including peroxidase (POX), superoxide dismutase (SOD), glutathione *S*-transferase (GST), and glutathione synthetase (GS), which were also targeted by the heat-responsive miRNAs.

### Gene Ontology Analysis

**FIGURE 6:**
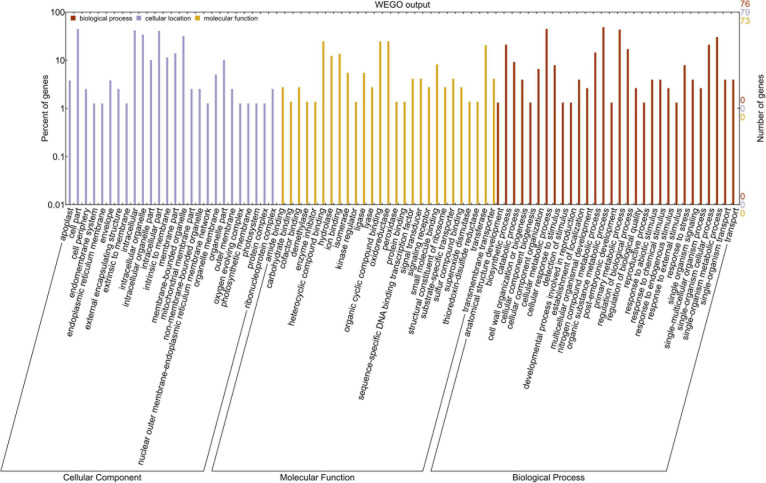
The gene ontology analysis of the heat-responsive miRNAs-targeted genes in wheat. The red, yellow, and blue bars symbolize biological process, molecular functions, and cellular components, respectively. The *x*-axis represents three GO categories; the right side of *y*-axis represents the number of miRNA target genes, while the left side of *y*-axis represents the percentage of the target genes.

Gene Ontology was conducted using WEGO to find the putative functions of miRNA-targeted genes expressed due to heat stress. These targeted genes were grouped into three classes: biological process (30 GO terms), molecular function (27 GO terms), and cellular component (28 GO terms) (Figure 6). GO analysis showed the role of these genes mainly in biosynthetic, metabolic, reproductive, and stress-responsive biological processes. Most of the genes were found to be associated with molecular functions such as carbohydrate binding, kinase regulator, oxidoreductase, peroxidase, DNA binding TF, signal transducer, SOD, thioredoxin disulfide reductase, transferase, and were found to be located mainly in the intracellular organelle part, apoplast, and in the membrane of the cell organelles (Figure 6). Therefore, the Gene Ontology analysis explained the role of miRNA-targeted genes in providing tolerance against terminal heat stress. The calculated *p*-values for GO terms are given in Supplementary Table 4.

### Correlation of Trait and Genotypic Data

In order to understand the correlation between genotypic and trait data of five heat-tolerance-related traits, efforts have been made to analyze genotypic data of two most promising diagnostic markers (miR159c and miR165b) showing association with heat tolerance. It was noticed that marker “miR159c” showed the presence of allele in all the heat-tolerant genotypes, while it is absent in heat-susceptible genotypes. Similarly, marker “miR165b” showed the absence of alleles in heat-tolerant genotypes, while it showed the presence of allele in heat-susceptible genotypes. The 100% specificity of alleles in these markers showing 100% association with heat tolerance is considered important, and this association was further confirmed by arranging trait data of heat-tolerant and heat-susceptible genotypes and testing their mean difference. The analysis of trait data of all the five traits (CTD, RWC, electrolyte leakage to test cell membrane stability, SPAD chlorophyll content, and proline content) recorded showed clear-cut mean difference between heat-tolerant vs. heat-susceptible genotypes. The heat-tolerant genotypes possessing specific alleles possess superior trait performance than heat-susceptible genotypes (Supplementary Table 5).

## Discussion

The environmental temperature increasing year by year due to the global warming emerged as a significant threat of terminal heat stress in wheat. Heat stress effect is more prominent when wheat was sown late in December instead of November due to delay in harvesting of wheat-preceding crop such as cotton and rice. Late-sown wheat experiences heat stress at the reproductive stages, which ultimately decreases the grain quality and productivity. In order to reduce the yield loss occurring due to excessive heat effect, there is a strong need to develop enhanced terminal heat-tolerant wheat varieties. Wheat plants also adapt to terminal heat stress by shortening the grain filling period, which causes a decrease in grain number, weight, and quality. Global wheat production is estimated to have decreased by 6% with every 1°C arise in temperature (Asseng et al., 2015).

Among the molecular markers, SSR markers were preferred for genetic diversity analysis, markers-assisted breeding, quantitative trait locus (QTL) mapping, etc., because of their distribution throughout the genome (Mir and Varshney, 2013; Mir et al., 2013; Kumar et al., 2021). Previous reports have revealed the function of miRNAs in controlling the heat stress, and a distinctive difference in miRNA families and their expression level was found in control and heat-stressed wheat genotypes (Kumar et al., 2015; Sailaja et al., 2017; Ravichandran et al., 2019; Rangan et al., 2020). A vast number of SSRs were reported within the protein-coding regions and their UTRs. However, the data on SSRs from non-coding regions of genes including miRNA genes in wheat genome are very limited. In addition, some miRNAs families are extremely conserved from millions of years. Therefore, SSRs derived from these miRNAs (miRNA-SSR) can better serve as a functional marker to differentiate closely related wheat genotypes as compared to previously known markers including RAPD, RFLP, and SSR (Ganie and Mondal, 2015; Tyagi et al., 2019, 2021). The use of miRNA-SSR markers for genetic diversity studies have become the hot topic during the last decade. Many studies have been conducted to differentiate terminal heat-tolerant wheat genotypes using SSR markers (Kumar et al., 2016; Hassan, 2016; Al-Ashkar et al., 2020). Yet to our knowledge, only two studies screened the miRNA-SSR for genetic diversity analysis and for characterization of a terminal heat-tolerant and heat-susceptible wheat genotypes (Sharma et al., 2021; Tyagi et al., 2021).

Polymorphism or PIC values were found to be important parameters to study the genetic diversity among genotypes. If the PIC value is >0.5, the marker is highly informative, and if the PIC values between 0.25 and 0.5, the marker is assumed moderately informative; therefore, markers with PIC value > 0.7 are considered appropriate for diversity studies and genetic mapping (Botstein et al., 1980; Bandelj et al., 2004). In this study, the 19 polymorphic miRNA-SSR markers showed a high polymorphism with averaged 2.9 alleles per locus and the averaged PIC value of 0.48, which was found to be higher than that reported by Sharma et al. (2021) (averaged 2.58 alleles/locus and PIC value of 0.35/locus), who screened 37 markers on 36 wheat genotypes, and lower than that reported by Tyagi et al. (2021) (averaged 3.4 alleles/locus, however, lower PIC value ranged from 0.16 to 0.38), who conducted genetic diversity studies using 13 miRNA-SSRs on 37 wheat genotypes in response to heat stress.

The dendrogram and structure analysis performed with 19 heat-responsive polymorphic miRNA-SSRs divided the population of 20 wheat genotypes into 3 clusters based on their genetic makeup. Sharma et al. (2021) clustered 36 wheat genotypes into 4 clusters using 37 cgSSR and miRNA-SSR markers, whereas Tyagi et al. (2021) divided 37 wheat genotypes into 4 clusters with 7 polymorphic miRNA-SSR markers. Both dendrogram and population structure results showed the presence of both heat-tolerant and heat-susceptible wheat genotypes into one cluster, which can be either due to their common ancestry or that an inadequate number of miRNA-SSRs was used for genetic diversity analysis. Similar results were obtained by Tyagi et al. (2021). However, the report of Sharma et al. (2021) distinctly categorized the heat stress-tolerant and heat stress-susceptible wheat genotypes. Moreover, the presence of heat-susceptible wheat genotypes into cluster I and heat-tolerant genotypes in cluster II highlighted the usefulness of these polymorphic markers in screening of wheat genotypes for their heat tolerance level.

The expression of heat-stress-responsive genes was found to be regulated by various miRNAs in response to heat stress. For example, an overexpression of miR156 was reported in *Arabidopsis*, as it generates memory in response to heat stress (Stief et al., 2014), whereas a downregulation of miR159 was noticed under heat stress, as it regulates the MYB TFs. In this study, the miRNAs (miR159c and 165b) were found to target the peroxidase, phenylalanine ammonia-lyase *(PAL)*, RING-type E3 ubiquitin transferase, Xyloglucan endotransglucosylase/hydrolase (XTH), etc., which provides heat tolerance to plants by the process antioxidant metabolism and phenolics accumulations and increased the expression of heat shock proteins by degrading the suppressor proteins of heat-responsive genes, respectively (Moura et al., 2017; Peng et al., 2019). Transgenic rice plants overexpressing miR159 were more susceptible to heat stress as compared to control plants (Wang et al., 2012). Yan et al. (2016) showed the downregulation of miR165/miR166 in *Arabidopsis*; these miRNAs target the ABA-responsive genes, which are key players in providing tolerance to abiotic stress. In addition, Ravichandran et al. (2019) revealed the role of heat-responsive miR528 and miR9662 miRNAs in wheat in regulating the antioxidants and mitochondrial proteins, respectively. Our study also identified many miRNAs-targeted genes codes for heat-responsive proteins, antioxidants, and TFs including ARF, WRKY, hsp70, hsp17, POX, SOD, GST, and GS. These results were also supported by earlier studies (Goswami et al., 2014; Ravichandran et al., 2019; Su et al., 2019; Rangan et al., 2020). Therefore, the identified miRNA-SSR markers (miR159c and miR165b) that are able to differentiate heat-tolerant and heat-susceptible wheat genotypes will play a significant role in breeding programs. Additional support was provided by trait data analysis of five most important traits (CTD, RWC, electrolyte leakage to test cell membrane stability, SPAD chlorophyll content, and proline content) related to heat tolerance. The analysis clearly indicated that the marker loci miR159c and miR165b showed linkage with heat tolerance and related five traits. The results indicated that these two markers could be used in wheat molecular breeding programs aimed at enhancing heat tolerance of wheat varieties for the development of next-generation heat-tolerant wheat varieties.

In conclusion, our study identified 19 polymorphic miRNA-SSRs markers, but among these, only two miRNA-SSR markers (miR159c and miR165b) were able to differentiate terminal heat-tolerant genotypes from the susceptible one. Therefore, the identified miRNA-SSR markers enable the studying of genetic diversity and MAB. As the physiological and phenotypic data are not sufficient to distinguish the tolerant and susceptible cultivars, the polymorphic miRNA-SSR markers will help the breeders to select terminal heat-tolerant wheat genotypes or marker-assisted selection at initial growth stages. This will further strengthen the release of new terminal heat-tolerant wheat varieties.

## Data Availability Statement

The original contributions presented in the study are included in the article/Supplementary Material, further inquiries can be directed to the corresponding author/s.

## Author Contributions

PS and VS conducted the experiments. PS and AK performed bioinformatics data analysis. PS drafted the manuscript. UK served as principal investigator, obtained funding for this research, and also revised the manuscript. OD, PB, and RM reviewed the manuscript. All authors agreed to the final manuscript.

## Conflict of Interest

The authors declare that the research was conducted in the absence of any commercial or financial relationships that could be construed as a potential conflict of interest.

## Publisher’s Note

All claims expressed in this article are solely those of the authors and do not necessarily represent those of their affiliated organizations, or those of the publisher, the editors and the reviewers. Any product that may be evaluated in this article, or claim that may be made by its manufacturer, is not guaranteed or endorsed by the publisher.

## Footnotes

1
http://www.mirbase.org/

2
https://plants.ensembl.org/

3
https://wheat.pw.usda.gov/demos/BatchPrimer3/0

4
https://gene-calc.pl/

5
http://taylor0.biology.ucla.edu/structureHarvester/

6
http://plantgrn.noble.org/psRNATarget/

7
https://biodb.swu.edu.cn/cgi-bin/wego/index.pl
