# Supplementary material for: Discovery of miRNAs and Development of Heat-Responsive miRNA-SSR Markers for Characterization of Wheat Germplasm for Terminal Heat Tolerance Breeding
Source: Front Genet. 2021 Jul 28;12:699420. doi: 10.3389/fgene.2021.699420 (PMC8356722; doi:10.3389/fgene.2021.699420)
Supplement: Supplementary file 1 [file Table_1.docx]

**Supplementary Table S1.** Details of heat responsive miRNAs used in this study

| **S. No.** | **miRNA** | **miRNA ID** | **miRNA Mature Sequence** |
| --- | --- | --- | --- |
| 1 | miR156 | MIMAT0018208 | UGACAGAAGAGAGUGAGCACA |
| 2 | miR156h | MIMAT0031156 | UGACAGAAGAGAGUGAGCAC |
| 3 | miR159 | MIMAT0001468 | UUUGGAUUGAAGGGAGCUCUG |
| 4 | miR159a | MIMAT0005343 | UUUGGAUUGAAGGGAGCUCUG |
| 5 | miR159b | MIMAT0005344 | UUUGGAUUGAAGGGAGCUCUG |
| 6 | miR159c | MIMAT0001024 | AUUGGAUUGAAGGGAGCUCCA |
| 7 | miR159f | MIMAT0001027 | CUUGGAUUGAAGGGAGCUCUA |
| 8 | miR160 | MIMAT0005345 | UGCCUGGCUCCCUGUAUGCCA |
| 9 | miR160a | MIMAT0037156 | UGCCUGGCUCCCUGUAUGCCA |
| 10 | miR164 | MIMAT0005346 | UGGAGAAGCAGGGCACGUGCA |
| 11 | miR164a | MIMAT0000633 | UGGAGAAGCAGGGCACGUGCA |
| 12 | miR165a | MIMAT0000187 | UCGGACCAGGCUUCAUCCCCC |
| 13 | miR165b | MIMAT0000188 | UCGGACCAGGCUUCAUCCCCC |
| 14 | miR166 | MIMAT0022882 | GGAAUGUUGGCUGGCUCGAGG |
| 15 | miR166d | MIMAT0000192 | UCGGACCAGGCUUCAUUCCCC |
| 16 | miR166f | MIMAT0000640 | UCGGACCAGGCUUCAUUCCCC |
| 17 | miR166j | MIMAT0022887 | GAAUGACGUCCGGUCUGAAGA |
| 18 | miR166k | MIMAT0022870 | GGUUUGUUGUCUGGCUCGAGG |
| 19 | miR167a | MIMAT0037092 | UGAAGCUGCCAGCAUGAUCUA |
| 20 | miR167b | MIMAT0000197 | UGAAGCUGCCAGCAUGAUCUA |
| 21 | miR167c | MIMAT0035789 | UGAAGCUGCCAGCAUGAUCUGC |
| 22 | miR167d | MIMAT0001039 | UGAAGCUGCCAGCAUGAUCUG |
| 23 | miR168 | MIMAT0018216 | GAUCCCGCCUUGCACCAAGUGAAU |
| 24 | miR169 | MIMAT0018217 | GUAGCCAAGGAUGAAUUGCCA |
| 25 | miR171 | MIMAT0031888 | UAUUGGCCUGGUUCACUCAGA |
| 26 | miR171a | MIMAT0005348 | UGAUUGAGCCGUGCCAAUAUC |
| 27 | miR171b | MIMAT0018227 | UUGAGCCGUGCCAAUAUCACG |
| 28 | miR172a | MIMAT0001069 | AGAAUCUUGAUGAUGCUGCAU |
| 29 | miR172c | MIMAT0001071 | UGAAUCUUGAUGAUGCUGCAC |
| 30 | miR172d | MIMAT0022885 | GCAGCACCAUCAAGAUUCAC |
| 31 | miR319 | MIMAT0018211 | UUGGACUGAAGGGAGCUCCCU |
| 32 | miR393a | MIMAT0000957 | UCCAAAGGGAUCGCAUUGAUC |
| 33 | miR395a | MIMAT0037163 | UGAAGUGUUUGGGGGAACUC |
| 34 | miR396d | MIMAT0037160 | UCCACAGGCUUUCUUGAACUG |
| 35 | miR396e | MIMAT0001601 | UCCACAGGCUUUCUUGAACUG |
| 36 | miR397 | MIMAT0035795 | UCACCGGCGCUGCACACAAUG |
| 37 | miR398 | MIMAT0018225 | UGUGUUCUCAGGUCGCCCCCG |
| 38 | miR398c | MIMAT0031912 | AGGGUUGAUAUGAGAACACAC |
| 39 | miR399a | MIMAT0037240 | GGGCGCUUCUCCAUUGGCACGG |
| 40 | miR400 | MIMAT0001001 | UAUGAGAGUAUUAUAAGUCAC |
| 41 | miR404 | MIMAT0001005 | AUUAACGCUGGCGGUUGCGGCAGC |
| 42 | miR408 | MIMAT0037188 | CAGGGAUGGAGCAGAGCAAGG |
| 43 | miR408a | MIMAT0001748 | CUGCACUGCCUCUUCCCUGGC |
| 44 | miR528 | MIMAT0037192 | UGGAAGGGGCAUGCAGAGGAG |
| 45 | miR824 | MIMAT0004277 | UAGACCAUUUGUGAGAAGGGA |
| 46 | miR829 | MIMAT0032019 | ACUUUGAAGCUUUGAUUUGAA |
| 47 | miR830 | MIMAT0004247 | UCUUCUCCAAAUAGUUUAGGUU |
| 48 | miR845b | MIMAT0004317 | UCGCUCUGAUACCAAAUUGAUG |
| 49 | miR863 | MIMAT0004309 | UUAUGUCUUGUUGAUCUCAAU |
| 50 | miR1118 | MIMAT0005353 | CACUACAUUAUGGAAUGGAGGGA |
| 51 | miR1128 | MIMAT0005363 | UACUACUCCCUCCGUCCGAAA |
| 52 | miR1130a | MIMAT0005365 | CCUCCGUCUCGUAAUGUAAGACG |
| 53 | miR1137a | MIMAT0005372 | UAGUACAAAGUUGAGUCAUC |
| 54 | miR1318 | MIMAT0022950 | CAGGUGUCAUCUCCCCUGAAC |
| 55 | miR1432 | MIMAT0037214 | UCAGGAGAGAUGACACCGACG |
| 56 | miR1848 | MIMAT0007768 | CCUCGCCGGCGCGCGCGUGCA |
| 57 | miR2096 | MIMAT0010056 | UGCCGAUUUCCCCCUCGGGCG |
| 58 | miR2102 | MIMAT0010068 | GGGCAAGCCGCCGCCGCCAC |
| 59 | miR2111b | MIMAT0011152 | UAAUCUGCAUCCUGAGGUUUA |
| 60 | miR2122 | MIMAT0011182 | UUUCAAAAAUAACCUUUUGUUC |
| 61 | miR5072 | MIMAT0020552 | CGAUUCCCCAGCGGAGUCGCCA |
| 62 | miR5077 | MIMAT0020574 | GUUCGCGUCGGGUUCACCA |
| 63 | miR5144 | MIMAT0021088 | UUCUUGUGCUGCUGAAGAGAC |
| 64 | miR5384 | MIMAT0035797 | UGAGCGCGCCGCCGUCGAAUG |
| 65 | miR5384a | MIMAT0021682 | CGCGCCGCCGUCCAGCGG |
| 66 | miR5386 | MIMAT0021684 | CGUCGCUGUCGCGCGCGCUG |
| 67 | miR9662a | MIMAT0035772 | UUGAACAUCCCAGAGCCACCG |
| 68 | miR9662b | MIMAT0035780 | UGAACAUCCCAGAGCCACCGG |
| 69 | miR9664 | MIMAT0035775 | UUGCAGUCCUCGAUGUCGUAG |
| 70 | miR9772 | MIMAT0036982 | UGAGAUGAGAUUACCCCAUAC |
| 71 | miR857 | MIMAT0004301 | UUUUGUAUGUUGAAGGUGUAU |
| 72 | miR865 | MIMAT0004314 | UUUUUCCUCAAAUUUAUCCAA |
| 73 | miR835 | MIMAT0004255 | UUCUUGCAUAUGUUCUUUAUC |
| 74 | miR394 | MIMAT0000958 | UUGGCAUUCUGUCCACCUCC |
| 75 | miR1117 | MIMAT0005352 | UAGUACCGGUUCGUGGCACGAACC |
| 76 | miR1122a | MIMAT0005357 | UAGAUACAUCCGUAUCUAGA |
| 77 | miR1125 | MIMAT0005360 | AACCAACGAGACCAACUGCGGCGG |
| 78 | miR1121 | MIMAT0005356 | AGUAGUGAUCUAAACGCUCUUA |
| 79 | miR1133 | MIMAT0005368 | CAUAUACUCCCUCCGUCCGAAA |
| 80 | miR1135 | MIMAT0005370 | CUGCGACAAGUAAUUCCGAACGGA |
| 81 | miR1136 | MIMAT0005371 | UUGUCGCAGGUAUGGAUGUAUCUA |
| 82 | miR530 | MIMAT0036987 | UGCAGUGGCAUAUGCAACUCU |
| 83 | miR1123 | MIMAT0005358 | UCCGUGAGACCUGGUCUCAUAGA |
| 84 | miR1439 | MIMAT0005993 | UUUUGGAACGGAGUGAGUAUU |
| 85 | miR3462 | MIMAT0017777 | UUGUCCCGGCAUCCCGAACGU |
| 86 | miR5652 | MIMAT0022424 | UUGAAUGUGAAUGAAUCGGGC |
| 87 | miR5062 | MIMAT0035781 | UGAACCUUAGGGAACAGCCGCAU |
| 88 | miR5064 | MIMAT0020571 | CGAAUUUGUCCAUAGCAUCAG |
| 89 | miR5071 | MIMAT0020551 | UCAAGCAUCAUAUCGUGGACA |
| 90 | miR5073 | MIMAT0020555 | GUUUGGUGAAUCGGAAACUAUUU |
| 91 | miR5084 | MIMAT0020566 | AUACAGUACUGCAGAGGAUCCUAA |
| 92 | miR5538 | MIMAT0022174 | ACUGAACUCAAUCACUUGCUGC |
| 93 | miR1885a | MIMAT0009213 | CAUCAAUGAAAGGUAUGAUUCC |
| 94 | miR9669 | MIMAT0035783 | UACUGUGGGCACUUAUUUGAC |
| 95 | miR9670 | MIMAT0035785 | AGGUGGAAUACUUGAAGAAGA |
| 96 | miR1131 | MIMAT0005366 | UAGUACCGGUUCGUGGCUAACC |
| 97 | miR846 | MIMAT0032023 | CAUUCAAGGACUUCUAUUCAG |
| 98 | miR842 | MIMAT0004264 | UCAUGGUCAGAUCCGUCAUCC |
| 99 | miR823 | MIMAT0004240 | UGGGUGGUGAUCAUAUAAGAU |
| 100 | miR6941 | - | NA |
| 101 | miR3182 | - | NA |
| 102 | miR2012 | - | NA |
| 103 | miR2020 | - | NA |
| 104 | miR2006 | - | NA |
