# Supplementary material for: Discovery of miRNAs and Development of Heat-Responsive miRNA-SSR Markers for Characterization of Wheat Germplasm for Terminal Heat Tolerance Breeding
Source: Front Genet. 2021 Jul 28;12:699420. doi: 10.3389/fgene.2021.699420 (PMC8356722; doi:10.3389/fgene.2021.699420)
Supplement: Supplementary file 2 [file Table_2.docx]

**Supplementary Table S2.** List of miRNA-SSR markers used for molecular diversity analysis in selected wheat genotypes

| **S.**  **No.** | **Marker Name** | **Forward Primer Sequence (5’–3’)** | **Reverse Primer Sequence (5’–3’)** | **Tm (°C)** | **Product Size** | **Monomorphic/**  **Polymorphic** |
| --- | --- | --- | --- | --- | --- | --- |
| 1 | miR156 | ATCTTTCTCTCTCCGCTTCC | GCATCCATCCGTCCTCTTTA | 54 | 178 | Monomorphic |
| 2 | miR 156h | TGCGTACATGGGTTTGATCT | GCATCCATCCGTCCTCTTTA | 52 | 196 | Monomorphic |
| 3 | miR159 | CTCACCCCTCTATAAAACGAC | CTACATCTATGGGGCTAGGAG | 50 | 140-260 | **Polymorphic** |
| 4 | miR159a | CCCCCTTCTTACCCCTCTAT | CTCACAAGCCAAAGCCAAG | 54.5 | 198 | Monomorphic |
| 5 | miR159b | ATTTTCCTTTCAATGACACCT | AAGAGATGGAACGGAAACTAC | 51 | 165-169 | **Polymorphic** |
| 6 | miR159c | CTTTCCCTCGTGCTTGGAT | GCATAGTGATTTGATTTTCTTGTTAGC | 52 | 180-250 | **Polymorphic** |
| 7 | miR159f | ACCTGTATAGGTTTTGCATGA | TTAGGTGCAGACTGAAAACAT | 51.5 | 130-162 | **Polymorphic** |
| 8 | miR160 | TATATGCACCGATGGCACAG | CGAAACAGCTCCACAATTCA | 53.5 | 173 | Monomorphic |
| 9 | miR160a | CAGCTTAGCCGTGTATGGAA | ATGACTCCCTCAATGCCAAC | 53.5 | 174 | Monomorphic |
| 10 | miR164 | TCACTGGGAAAAGGGACAGA | ACCGTGATGGAGGAGAAGAA | NA | NA | NA |
| 11 | miR164a | ACTGCACTGCACGTGTTCTT | TTGAAGACGCATACCTCGTG | 59.5 | 135-315 | **Polymorphic** |
| 12 | miR165a | CATGTGTATGTGTGAATGGTC | TAGGATGCTGGAGTAACAGAA | NA | NA | NA |
| 13 | miR165b | CAACGGTGTGATTGTAAAAA | CGAAGTTTAATTTGGTTATGC | 50 | 146-280 | **Polymorphic** |
| 14 | miR166 | GGATCCAGGTTGGTTAATCG | TATACGCATGCCACCAAAAA | 59 | 172 | Monomorphic |
| 15 | miR166d | GTCATCTTCCTCCTCTCTCTC | ATCATTACACCAATCTGCATC | 52 | 132 | Monomorphic |
| 16 | miR166f | GCACTTCACAGTGCTTTATTT | CAACAGACGGACAAAAATAGT | NA | NA | NA |
| 17 | miR166j | CAGAGAGTAGACGTGCGTAGA | GAGCAAGATGACCTTAACAGA | 52.5 | 173 | Monomorphic |
| 18 | miR166k | GATCTATCTGGCTGTCCTCTC | ACAAACTGAGCATATGTGGAC | 52.5 | 195 | Monomorphic |
| 19 | miR167a | AGGCCGCCATACAGAAGATA | TTCAGTCTCTCACACCAGCAA | 56 | 165 | Monomorphic |
| 20 | miR167b | ATATGAGGCCGCCATACAGA | ATGCTGGACGAGCAGATCAT | 56 | 171 | Monomorphic |
| 21 | miR167c | CCATCACTCCTCACTCATTCA | AAGCTCCTTACCGTGGTTCA | 53 | 180 | Monomorphic |
| 22 | miR167d | GGAGCACAACGAAAACCCTA | CGAAAGAAAAGAACCAAGACAGA | 54 | 205 | Monomorphic |
| 23 | miR168 | CCTTGCACCAAGTGAATC | CGGCAGCGAGTAGTTAGTAAT | 52 | 161 | Monomorphic |
| 24 | miR169 | AGACAAAGTGTGAGCAGAAAG | ACACAATGATAAACTGGCATC | 52 | 151 | Monomorphic |
| 25 | miR171 | GGAGTGAGCCATGTAGCA | TTCCTTCTCCGATCACAA | 50 | 146 | Monomorphic |
| 26 | miR171a | GTCGGGGGACTTGAATGC | GGGAGAAGAGAGAGGGGAGA | NA | NA | NA |
| 27 | miR171b | CTGAACGCTACTGAGCCACT | CTACCAACACGGCAGCACTA | 55 | 178-300 | **Polymorphic** |
| 28 | miR172a | GCCTCTCTTTGTCTTGATCC | CCGACTGTGATCTGGTATCT | 53 | 170 | Monomorphic |
| 29 | miR172c | CCTCTCTTTGTCTTCATCCA | AAGAACCGACTGTGATCTGA | 51.5 | 130-180 | **Polymorphic** |
| 30 | miR172d | CAGGAGGGAGAAGAGATAGAT | CAATGGATCAAGACAAAGAGA | 52 | 158 | Monomorphic |
| 31 | miR319 | AGAGCCTTTTTCTTGTGCTG | CACCGTAGCCTGTGTATTCTAAA | 54 | 178 | Monomorphic |
| 32 | miR393a | CCTATATAAGGACCTCACTGGA | AGGCATTGTTGCTCTCTCT | 52 | 157-175 | **Polymorphic** |
| 33 | miR395a | ACGGGAGATGAGCGGAGT | GTTGATTGCCACCCAGAGTT | 55 | 182 | Monomorphic |
| 34 | miR396d | AAGTTATATCGGACCGTGTG | AGGAAGGGGTCGTATAAATAG | 52 | 150-470 | **Polymorphic** |
| 35 | miR396e | GGGTTATATAAGCAGCGTGA | GAGAAGTTCAAGAAAGCTGTG | 51.5 | 176 | Monomorphic |
| 36 | miR397 | CTGCAGTACAACGAGCTG | AGCGATCGATTCTACTTATAGC | 52 | 150 | Monomorphic |
| 37 | miR398 | GCTTGATTGACGAGCGACT | CTGGAAGGTTGGAGTTGGTG | 55 | 241 | Monomorphic |
| 38 | miR398c | GGTCAAACAACAACACGAG | TGAAAACTGAGATGCGTGTA | 51 | 173 | Monomorphic |
| 39 | miR399a | GTCTCTGGCGAGTGAGATAG | AATCTCCTTTGGCAGATAGAC | 51 | 146 | **Polymorphic** |
| 40 | miR400 | CAATTGGAAGTTTCTCTACGA | CGAAGTCCATTGAAGATATGA | NA | NA | NA |
| 41 | miR404 | CTAAACCGGATAAAGGGTAGA | CAGAGGAACGCACGTAGT | 52 | 140-162 | **Polymorphic** |
| 42 | miR408 | CAACTGCTTCCTTCCACTGA | TCCCTCCGTTCCAAAATAAA | NA | NA | NA |
| 43 | miR408a | TGTAATGTGGATCTGATGTTG | CTAGCGGTCACCAATTTATC | 50 | 148 | Monomorphic |
| 44 | miR528 | TACTGCCACCAACCTGAACC | AAACACCCAAGCACACACAC | 55 | 185 | Monomorphic |
| 45 | miR824 | GGCCTTTGACTGAATTAGTGT | CCAGAAAGGAATTATTTTGGA | 50 | 134 | Monomorphic |
| 46 | miR829 | TATGATGCAACAAACAACAAG | AGACGTACTTCCCTCAAAAGT | 50 | 149 | Monomorphic |
| 47 | miR830 | AGTACGCCTTGATCTCCTCT | CTACGTTACCTTCCTCTCTCC | 53.5 | 130-320 | **Polymorphic** |
| 48 | miR845b | CCCTCTAGATTGGATCTCTCT | GGTCGGAGTTATGGATCTC | 52 | 145 | Monomorphic |
| 49 | miR857 | TTCTTGCCTACTTGTTTCTG | GTCGCCGTCTTTGAATTT | 50 | 267-272 | **Polymorphic** |
| 50 | miR863 | GCAAATTTGGAATTACTTGG | TAGTGTTGCTGTTGCAATAAA | 50 | 173 | Monomorphic |
| 51 | miR1118 | GTCTCTCAAATCATGACAAGC | AGCTTTAAGAGAGTGTTGCAC | 52 | 156 | Monomorphic |
| 52 | miR1128 | CACATCAAAATCTCCAGAAAG | TTTGGATGCTATATACCTCTTG | 50.5 | 154 | Monomorphic |
| 53 | miR1130a | AGTTGCACTGCTACAAGCTAC | ACTTTCGGATGTACTGTTCTG | 52 | 146-165 | **Polymorphic** |
| 54 | miR1137a | CCACACAATGGCAAGAAC | TCTGAAACTGAAACACTGGAC | 51 | 143 | Monomorphic |
| 55 | miR1318 | GCACAGGTGATAATGCAAC | GTGTATGAGCTCCAACATCTG | 52 | 162 | Monomorphic |
| 56 | miR1432 | TCTTATGGGGTATATCGAAGG | GGCTGGTGTCTGATTGTC | 52 | 153 | Monomorphic |
| 57 | miR1848 | CTTTCCCTCTTCTAGCAACAC | ATCTGGGACGAAGAACACTAC | 50 | 221 | Monomorphic |
| 58 | miR2096 | CTCATCATCTCCTTCCTCCT | GGAGATTCTCATCTCATCCTC | 50 | 200 | Monomorphic |
| 59 | miR2102 | ACCGCTGCTGTTGTATTG | TCAAGTCCTCTGCAAACAC | 52 | 167-175 | **Polymorphic** |
| 60 | miR2111b | CCATCCAACAATAACAACAAC | GTGGGCGATAATGTAGAAGAT | 50 | 151 | Monomorphic |
| 61 | miR2122 | GGGTGGACAGTAAAATCAGA | CCATTTTTCAGGATCATTCTT | 50 | 157-162 | **Polymorphic** |
| 62 | miR5072 | AGCCGATATCTCATCAAGACT | TGTGTCAACACACTTTTTGG | 51.5 | 138 | Monomorphic |
| 63 | miR5077 | GGGTGGGGAATTGCGTAG | CAGAACAGAGCCCACATACT | 55 | 152 | Monomorphic |
| 64 | miR5144 | GAGAGGAGGTGGATGGAG | CGAGACCACAAGCAAGTC | 52 | 299 | Monomorphic |
| 65 | miR5384 | AGGGGATCCTCCTCAGAT | AGGTGGTTGGTGGCCAAG | 52 | 280-350 | **Polymorphic** |
| 66 | miR5384a | CAATGGATGGCTACAAAATAG | ATGCGTACCGGATTCTGAT | 50 | 134 | Monomorphic |
| 67 | miR5386 | AAAGATTGACCTGAATGCAC | GTCAGTGAACATGAGCAAGAT | 51.5 | 152 | Monomorphic |
| 68 | miR9662a | CTTCACCAAACCCTCCTC | GAGATCCAGCAGAAGGAGA | 53 | 250 | Monomorphic |
| 69 | miR9662b | CCTTCACCAAACCCTCTT | GAGATCCAGCAGAAGGAGAT | 53 | 227-235 | **Polymorphic** |
| 70 | miR9664 | AAAATTAAGCTTGTCCCAAGT | ATTCACGAGTGAGCAGAGTAA | 50.5 | 157 | Monomorphic |
