# Supplementary material for: Discovery of miRNAs and Development of Heat-Responsive miRNA-SSR Markers for Characterization of Wheat Germplasm for Terminal Heat Tolerance Breeding
Source: Front Genet. 2021 Jul 28;12:699420. doi: 10.3389/fgene.2021.699420 (PMC8356722; doi:10.3389/fgene.2021.699420)
Supplement: Supplementary file 3 [file Table_3.docx]

**Supplementary Table S3.** Targets of the heat responsive miRNAs in wheat genome

| **S.No.** | **miRNA** | **miRNA Targets** | **Mode of Action** |
| --- | --- | --- | --- |
| 1 | miR156 | Xyloglucan endotransglucosylase/hydrolase, Endoglucanase, Beta-carotene hydroxylase B2, Glycerol-3-phosphate dehydrogenase, Peroxidase | Cleavage, translation |
| 2 | miR159 | Phenylalanine ammonia-lyase, Serine/threonine-protein kinase **,** RING-type E3 ubiquitin transferase, Peroxidase | Cleavage |
| 3 | miR159a | Phenylalanine ammonia-lyase, Phospholipase, Peroxidase | Cleavage, translation |
| 4 | miR160 | Auxin response factor, Glycosyltransferase, RING-type E3 ubiquitin transferase, Peroxidase, Glutamate receptor | Cleavage, translation |
| 5 | miR164 | NAC transcription factor 6A, Mitogen-activated protein kinase, Phytosulfokine-alpha 1**,** Histone H4 variant TH011, Oxygen evolving enhancer protein | Cleavage, translation |
| 6 | miR165 | Alpha-1,4 glucan phosphorylase, , Auxin response factor, Dirigent protein, Peroxidase, Glycosyltransferase, Peptidylprolyl isomerase, Histone deacetylase, Calcium-transporting ATPase, Xyloglucan endotransglucosylase/hydrolase | Cleavage |
| 7 | miR166 | Alpha-1,4 glucan phosphorylase, Beta-galactosidase, Auxin response factor, Glycosyltransferase, Beta-galactosidase, Peroxidase | Cleavage |
| 8 | miR167 | WRKY transcription factor 1, Auxin response factor, Peroxidase, RING-type E3 ubiquitin transferase, Flavin-containing monooxygenase, Glycosyltransferase, Exocyst subunit Exo70 family protein | Cleavage |
| 9 | miR167d | Glycosyltransferase, Peroxidase, Auxin response factor, Xyloglucan endotransglucosylase/hydrolase, Thioredoxin reductase, Glutathione S-transferase | Cleavage |
| 10 | miR168 | Beta-amylase, Chlorophyll a-b binding protein, Mitogen-activated protein kinase, Phenylalanine ammonia-lyase, Ferredoxin--NADP reductase, Sucrose synthase | Cleavage, translation |
| 11 | miR169 | Exocyst subunit Exo70 family protein, Alpha-1,4 glucan phosphorylase | Cleavage |
| 12 | miR171 | Glycosyltransferase, R1R2R3-MYB protein, Glycosyltransferase, Isocitrate dehydrogenase [NAD] subunit, Zinc finger protein, Methionine S-methyltransferase | CCleavage |
| 13 | miR172 | Serine/threonine-protein kinase, RING-type E3 ubiquitin transferase, Exocyst subunit Exo70 family protein, Flavin-containing monooxygenase, Superoxide dismutase [Cu-Zn], Cyclin-dependent kinase inhibitor, Pectin acetylesterase | Cleavage |
| 14 | miR172a | Glycosyltransferase, Flavin-containing monooxygenase, cyclin-dependent kinase inhibitor | Cleavage, translation |
| 15 | miR172d | Floral homeotic protein, Phytochrome, Glycosyltransferase, Auxin response factor, CTP synthase, Peptidylprolyl isomerase, Flavin-containing monooxygenase, Cyclin-dependent kinase inhibitor | Cleavage |
| 16 | mir319 | Peroxidase, Phenylalanine ammonia-lyase | Cleavage, translation |
| 17 | miR393a | Cellulose synthase | Cleavage |
| 18 | miR395a | Ribosomal protein L19, Mitochondrial Rho GTPase ,Flavin-containing monooxygenase, Exocyst subunit Exo70 family protein | cleavage |
| 19 | miR396 | Growth regulating factor 7-D1/A1/B1, Growth regulating factor 5-A1/D1,GRF 1-D1/A1/B1, Glutamate dehydrogenase, Chalcone-flavonone isomerase family protein, Histone H3.2 | Cleavage |
| 20 | miR397 | Potassium transporter, Dehydrin, Beta-galactosidase | Cleavage |
| 21 | miR398 | 3-ketoacyl-CoA synthase, RING-type E3 ubiquitin transferase, Chloride channel (CLC) protein, Endoglucanase | Cleavage, translation |
| 22 | miR398c | Serine/threonine-protein kinase, Sodium/hydrogen exchanger, Glucose-6-phosphate 1-dehydrogenase, Phytochrome, Dirigent protein, Growth regulating factor 8-B1 | Cleavage |
| 23 | miR399a | Dirigent protein | Cleavage |
| 24 | miR400 | Acetyl-coenzyme A synthetase, Cytochrome P450 | Cleavage |
| 25 | miR404 | Exocyst subunit Exo70 family protein, | Cleavage |
| 26 | miR408 | Histone H4, Glycosyltransferase, Histone H2A, Chlorophyll a-b binding protein | Cleavage |
| 27 | miR528 | Laccase,Peroxidase,Glycosyltransferase,Replication protein A subunit,Endoglucanase,Auxin response factor,Phospholipase,Citrate synthase,CTP synthase | Translation, Cleavage |
| 28 | miR824 | fructan 6-fructosyltransferase, Superoxide dismutase, Beta-galactosidase, Sucrose synthase, Peroxidase, Xyloglucan endotransglucosylase/hydrolase | Cleavage, translation |
| 29 | miR829 | Exocyst subunit Exo70 family protein, Phospholipase D, Glycosyltransferase, Auxin-responsive protein | Cleavage, translation |
| 30 | miR830 | NADPH-dependent diflavin oxidoreductase 1, E3 ubiquitin-protein ligase, Diacylglycerol kinase, Exocyst subunit Exo70 family protein | Cleavage, translation |
| 31 | miR845 | RPS13 description:Rp13(Ribosomal protein S13), NADPH-protochlorophyllide oxidoreductase | Cleavage |
| 32 | miR857 | Cytosolic acetyl-CoA carboxylase, ATP-dependent 6-phosphofructokinase, Laccase, Glucose-1-phosphate adenylyltransferase, Hexosyltransferase, Pyrophosphate--fructose 6-phosphate 1-phosphotransferase subunit beta | Cleavage |
| 33 | miR863 | Sucrose synthase, Glucose-6-phosphate 1-epimerase, Dirigent protein, Peroxidase, Thioredoxin, Replication protein A subunit, Peroxidase | Cleavage, translation |
| 34 | miR1118 | Glycosyltransferase, 5-methylcytosine DNA glycosylase | Cleavage, translation |
| 35 | miR1128 | Exocyst subunit Exo70 family protein, Serine/threonine-protein kinase, Starch synthase, Beta-galactosidase, Bidirectional sugar transporter SWEET, Xyloglucan endotransglucosylase/hydrolase, Glutathione synthetase | Cleavage |
| 36 | miR1130a | Peroxidase, E3 ubiquitin protein ligase, biosynthesis monooxygenase COQ6, Glycosyltransferases, Ferrochelatase | Cleavage, translation |
| 37 | miR1137a | Starch synthase, 60S ribosomal protein L36, Xyloglucan endotransglucosylase/hydrolase, Cellulose synthase, Glutamate receptor, RBR-type E3 ubiquitin transferase, Glycosyltransferase, Nitrate reductase | Cleavage |
| 38 | miR1318 | No target found | NA |
| 39 | miR1432 | Beta-galactosidase, AKT1-like potassium channel, Trehalose 6-phosphate phosphatase, Endoglucanase | Cleavage |
| 40 | miR1848 | Mitogen-activated protein kinase | Cleavage |
| 41 | miR2096 | RING-type E3 ubiquitin transferase, Protein transport protein SEC23, Ethylene responsive transcription factor 5a (ERF 5A) | Cleavage |
| 42 | miR2102 | CTP synthase | Cleavage |
| 43 | miR2111b | Glycosyltransferase, Dirigent protein, Fatty acyl-CoA reductase, CTP synthase | Cleavage, translation |
| 44 | miR2122 | RBR-type E3 ubiquitin transferase, ATP-dependent 6-phosphofructokinase, Phenylalanine ammonia-lyase, Cellulose synthase, E3 ubiquitin-protein ligase | Cleavage |
| 45 | miR5072 | Glucose-6-phosphate 1-dehydrogenase, Outer membrane channel protein OEP16-2, RING-type E3 ubiquitin transferase | Cleavage |
| 46 | miR5077 | Protein disulfide-isomerase, Fatty acyl-CoA reductase, Cinnamoyl-CoA reductase, CASP-like protein, Cellulose synthase | Cleavage |
| 47 | miR5144 | Exocyst subunit Exo70 family protein, 50S ribosomal protein L31, Peptidylprolylisomerise**,** Glycosyltransferase, Phytochrome, NADPH--cytochrome P450 reductase, Malic enzyme | Cleavage, translation |
| 48 | miR5384 | 3-ketoacyl-CoA synthase, CASP-like protein, Exocyst subunit Exo70 family protein, Xylanase inhibitor TAXI-IV, Phospholipase D, Glycosyltransferase, Auxin efflux carrier component, Pyruvate kinase | Cleavage |
| 49 | miR5386 | No target found | NA |
| 50 | miR9662 | No target found | NA |
| 51 | miR9664 | Nucleotide binding-leucine rich repeat protein | Cleavage |
