# Supplementary material for: Discovery of miRNAs and Development of Heat-Responsive miRNA-SSR Markers for Characterization of Wheat Germplasm for Terminal Heat Tolerance Breeding
Source: Front Genet. 2021 Jul 28;12:699420. doi: 10.3389/fgene.2021.699420 (PMC8356722; doi:10.3389/fgene.2021.699420)
Supplement: Supplementary file 4 [file Table_4.docx]

**Supplementary Table S4.** The p-values of GO terms

| **GO term** | **Ontology** | **Description** | **Number in input list** | **Number in BG/Ref** | **p-value** | **FDR** |
| --- | --- | --- | --- | --- | --- | --- |
| GO:0044710 | P | single-organism metabolic process | 50 | 27869 | 3.40E-10 | 8.50E-07 |
| GO:0044763 | P | single-organism cellular process | 58 | 40376 | 1.40E-08 | 1.80E-05 |
| GO:0046496 | P | nicotinamide nucleotide metabolic process | 11 | 1642 | 1.60E-07 | 5.70E-05 |
| GO:0072524 | P | pyridine-containing compound metabolic process | 11 | 1669 | 1.80E-07 | 5.70E-05 |
| GO:0019362 | P | pyridine nucleotide metabolic process | 11 | 1645 | 1.60E-07 | 5.70E-05 |
| GO:0043436 | P | oxoacid metabolic process | 26 | 10221 | 1.40E-07 | 5.70E-05 |
| GO:0019752 | P | carboxylic acid metabolic process | 25 | 9596 | 1.70E-07 | 5.70E-05 |
| GO:0006082 | P | organic acid metabolic process | 27 | 11065 | 1.70E-07 | 5.70E-05 |
| GO:0006732 | P | coenzyme metabolic process | 14 | 3094 | 3.20E-07 | 8.90E-05 |
| GO:0051186 | P | cofactor metabolic process | 16 | 4166 | 3.60E-07 | 9.10E-05 |
| GO:0032787 | P | monocarboxylic acid metabolic process | 21 | 7305 | 4.70E-07 | 0.00011 |
| GO:0044699 | P | single-organism process | 63 | 50975 | 5.70E-07 | 0.00012 |
| GO:0006733 | P | oxidoreduction coenzyme metabolic process | 12 | 2341 | 6.90E-07 | 0.00013 |
| GO:0006091 | P | generation of precursor metabolites and energy | 14 | 3330 | 7.70E-07 | 0.00014 |
| GO:0055114 | P | oxidation-reduction process | 23 | 9149 | 1.20E-06 | 0.0002 |
| GO:0044281 | P | small molecule metabolic process | 30 | 14959 | 1.80E-06 | 0.00029 |
| GO:0009987 | P | cellular process | 67 | 58786 | 2.10E-06 | 0.00031 |
| GO:0044711 | P | single-organism biosynthetic process | 31 | 16047 | 2.50E-06 | 0.00035 |
| GO:0006090 | P | pyruvate metabolic process | 10 | 1770 | 2.70E-06 | 0.00036 |
| GO:0071704 | P | organic substance metabolic process | 62 | 51371 | 3.30E-06 | 0.00041 |
| GO:0050896 | P | response to stimulus | 52 | 39029 | 7.80E-06 | 0.00084 |
| GO:0044262 | P | cellular carbohydrate metabolic process | 14 | 4044 | 7.20E-06 | 0.00084 |
| GO:0005984 | P | disaccharide metabolic process | 7 | 831 | 7.70E-06 | 0.00084 |
| GO:0005975 | P | carbohydrate metabolic process | 20 | 8137 | 1.00E-05 | 0.0011 |
| GO:0008152 | P | metabolic process | 65 | 57664 | 1.50E-05 | 0.0015 |
| GO:0044723 | P | single-organism carbohydrate metabolic process | 17 | 6266 | 1.60E-05 | 0.0015 |
| GO:0044237 | P | cellular metabolic process | 59 | 49214 | 2.00E-05 | 0.0019 |
| GO:0042221 | P | response to chemical | 37 | 23546 | 2.20E-05 | 0.0019 |
| GO:0005982 | P | starch metabolic process | 7 | 982 | 2.20E-05 | 0.0019 |
| GO:0016051 | P | carbohydrate biosynthetic process | 13 | 4030 | 3.30E-05 | 0.0027 |
| GO:0008610 | P | lipid biosynthetic process | 15 | 5283 | 3.40E-05 | 0.0027 |
| GO:0006739 | P | NADP metabolic process | 6 | 719 | 3.70E-05 | 0.0028 |
| GO:0009311 | P | oligosaccharide metabolic process | 7 | 1067 | 3.70E-05 | 0.0028 |
| GO:0019252 | P | starch biosynthetic process | 6 | 737 | 4.30E-05 | 0.0031 |
| GO:0006073 | P | cellular glucan metabolic process | 10 | 2605 | 7.30E-05 | 0.0051 |
| GO:0016052 | P | carbohydrate catabolic process | 9 | 2089 | 7.30E-05 | 0.0051 |
| GO:0044042 | P | glucan metabolic process | 10 | 2628 | 7.90E-05 | 0.0053 |
| GO:0009642 | P | response to light intensity | 8 | 1687 | 9.80E-05 | 0.0061 |
| GO:0019637 | P | organophosphate metabolic process | 15 | 5805 | 9.70E-05 | 0.0061 |
| GO:1901576 | P | organic substance biosynthetic process | 43 | 31446 | 9.30E-05 | 0.0061 |
| GO:0006790 | P | sulfur compound metabolic process | 11 | 3413 | 0.00015 | 0.0088 |
| GO:0044255 | P | cellular lipid metabolic process | 15 | 6058 | 0.00016 | 0.0092 |
| GO:0019693 | P | ribose phosphate metabolic process | 11 | 3479 | 0.00017 | 0.0097 |
| GO:0019748 | P | secondary metabolic process | 14 | 5410 | 0.00017 | 0.0097 |
| GO:0009117 | P | nucleotide metabolic process | 12 | 4105 | 0.00018 | 0.0097 |
| GO:0006753 | P | nucleoside phosphate metabolic process | 12 | 4119 | 0.00018 | 0.0098 |
| GO:0034637 | P | cellular carbohydrate biosynthetic process | 10 | 2953 | 0.0002 | 0.011 |
| GO:0046148 | P | pigment biosynthetic process | 8 | 1880 | 0.0002 | 0.011 |
| GO:0010207 | P | photosystem II assembly | 5 | 632 | 0.00022 | 0.011 |
| GO:0010035 | P | response to inorganic substance | 21 | 10992 | 0.00024 | 0.012 |
| GO:0009058 | P | biosynthetic process | 43 | 32675 | 0.00025 | 0.012 |
| GO:0010038 | P | response to metal ion | 12 | 4300 | 0.00027 | 0.013 |
| GO:0055086 | P | nucleobase-containing small molecule metabolic process | 12 | 4314 | 0.00028 | 0.013 |
| GO:0006098 | P | pentose-phosphate shunt | 5 | 687 | 0.00032 | 0.015 |
| GO:0009891 | P | positive regulation of biosynthetic process | 10 | 3175 | 0.00036 | 0.015 |
| GO:0009250 | P | glucan biosynthetic process | 8 | 2042 | 0.00035 | 0.015 |
| GO:0048511 | P | rhythmic process | 6 | 1096 | 0.00036 | 0.015 |
| GO:0006081 | P | cellular aldehyde metabolic process | 7 | 1549 | 0.00037 | 0.015 |
| GO:0051156 | P | glucose 6-phosphate metabolic process | 5 | 708 | 0.00037 | 0.015 |
| GO:0006629 | P | lipid metabolic process | 17 | 8205 | 0.00044 | 0.018 |
| GO:0006996 | P | organelle organization | 20 | 10672 | 0.00046 | 0.019 |
| GO:1901564 | P | organonitrogen compound metabolic process | 23 | 13333 | 0.0005 | 0.02 |
| GO:0042440 | P | pigment metabolic process | 8 | 2179 | 0.00054 | 0.021 |
| GO:0044264 | P | cellular polysaccharide metabolic process | 10 | 3343 | 0.00054 | 0.021 |
| GO:0031328 | P | positive regulation of cellular biosynthetic process | 9 | 2742 | 0.00054 | 0.021 |
| GO:0005976 | P | polysaccharide metabolic process | 11 | 4027 | 0.00059 | 0.022 |
| GO:0016114 | P | terpenoid biosynthetic process | 6 | 1214 | 0.00062 | 0.023 |
| GO:0019682 | P | glyceraldehyde-3-phosphate metabolic process | 6 | 1229 | 0.00066 | 0.024 |
| GO:0001666 | P | response to hypoxia | 5 | 814 | 0.00069 | 0.025 |
| GO:0015980 | P | energy derivation by oxidation of organic compounds | 5 | 828 | 0.00074 | 0.026 |
| GO:0036293 | P | response to decreased oxygen levels | 5 | 832 | 0.00076 | 0.026 |
| GO:0070482 | P | response to oxygen levels | 5 | 837 | 0.00078 | 0.027 |
| GO:0044249 | P | cellular biosynthetic process | 40 | 30853 | 0.00079 | 0.027 |
| GO:0044283 | P | small molecule biosynthetic process | 15 | 7111 | 0.00085 | 0.028 |
| GO:0044238 | P | primary metabolic process | 54 | 47561 | 0.00086 | 0.028 |
| GO:0006721 | P | terpenoid metabolic process | 6 | 1316 | 0.00093 | 0.03 |
| GO:0019684 | P | photosynthesis, light reaction | 6 | 1315 | 0.00093 | 0.03 |
| GO:0015979 | P | photosynthesis | 7 | 1877 | 0.0011 | 0.036 |
| GO:0009893 | P | positive regulation of metabolic process | 10 | 3712 | 0.0012 | 0.037 |
| GO:0043085 | P | positive regulation of catalytic activity | 5 | 920 | 0.0012 | 0.037 |
| GO:0046686 | P | response to cadmium ion | 9 | 3086 | 0.0012 | 0.037 |
| GO:0006950 | P | response to stress | 38 | 29274 | 0.0012 | 0.037 |
| GO:0010557 | P | positive regulation of macromolecule biosynthetic process | 8 | 2491 | 0.0013 | 0.038 |
| GO:0072330 | P | monocarboxylic acid biosynthetic process | 10 | 3758 | 0.0013 | 0.038 |
| GO:0008299 | P | isoprenoid biosynthetic process | 7 | 1928 | 0.0013 | 0.038 |
| GO:0001101 | P | response to acid chemical | 22 | 13354 | 0.0013 | 0.038 |
| GO:0051704 | P | multi-organism process | 25 | 16180 | 0.0014 | 0.039 |
| GO:0044724 | P | single-organism carbohydrate catabolic process | 6 | 1428 | 0.0014 | 0.04 |
| GO:0007623 | P | circadian rhythm | 5 | 971 | 0.0015 | 0.042 |
| GO:0006757 | P | ATP generation from ADP | 5 | 989 | 0.0016 | 0.042 |
| GO:0009135 | P | purine nucleoside diphosphate metabolic process | 5 | 997 | 0.0017 | 0.042 |
| GO:0006096 | P | glycolytic process | 5 | 989 | 0.0016 | 0.042 |
| GO:1901700 | P | response to oxygen-containing compound | 26 | 17327 | 0.0016 | 0.042 |
| GO:0046031 | P | ADP metabolic process | 5 | 997 | 0.0017 | 0.042 |
| GO:0009179 | P | purine ribonucleoside diphosphate metabolic process | 5 | 997 | 0.0017 | 0.042 |
| GO:0016053 | P | organic acid biosynthetic process | 13 | 6010 | 0.0016 | 0.042 |
| GO:0009185 | P | ribonucleoside diphosphate metabolic process | 5 | 997 | 0.0017 | 0.042 |
| GO:0031325 | P | positive regulation of cellular metabolic process | 9 | 3207 | 0.0016 | 0.042 |
| GO:0009056 | P | catabolic process | 21 | 12766 | 0.0018 | 0.044 |
| GO:0006165 | P | nucleoside diphosphate phosphorylation | 5 | 1015 | 0.0018 | 0.045 |
| GO:0009719 | P | response to endogenous stimulus | 24 | 15578 | 0.0018 | 0.045 |
| GO:0006720 | P | isoprenoid metabolic process | 7 | 2047 | 0.0018 | 0.045 |
| GO:0009132 | P | nucleoside diphosphate metabolic process | 5 | 1032 | 0.0019 | 0.047 |
| GO:0009725 | P | response to hormone | 23 | 14710 | 0.002 | 0.047 |
| GO:0007275 | P | multicellular organism development | 29 | 20549 | 0.002 | 0.048 |
| GO:0019725 | P | cellular homeostasis | 7 | 2087 | 0.002 | 0.048 |
| GO:0009416 | P | response to light stimulus | 15 | 7773 | 0.0021 | 0.048 |
| GO:0044767 | P | single-organism developmental process | 31 | 22628 | 0.0021 | 0.049 |
| GO:0046939 | P | nucleotide phosphorylation | 5 | 1060 | 0.0022 | 0.05 |
| GO:0016491 | F | oxidoreductase activity | 22 | 8456 | 1.30E-06 | 0.00032 |
| GO:0003824 | F | catalytic activity | 55 | 42216 | 4.70E-06 | 0.00059 |
| GO:0050660 | F | flavin adenine dinucleotide binding | 6 | 721 | 3.80E-05 | 0.0032 |
| GO:0016651 | F | oxidoreductase activity, acting on NAD(P)H | 7 | 1178 | 6.90E-05 | 0.0043 |
| GO:0048037 | F | cofactor binding | 10 | 2650 | 8.40E-05 | 0.0043 |
| GO:0050662 | F | coenzyme binding | 8 | 1716 | 0.00011 | 0.0046 |
| GO:1901265 | F | nucleoside phosphate binding | 28 | 17081 | 0.00022 | 0.0065 |
| GO:0000166 | F | nucleotide binding | 28 | 17081 | 0.00022 | 0.0065 |
| GO:1901363 | F | heterocyclic compound binding | 43 | 32889 | 0.0003 | 0.0065 |
| GO:0043167 | F | ion binding | 29 | 18287 | 0.00028 | 0.0065 |
| GO:0097159 | F | organic cyclic compound binding | 43 | 32929 | 0.00031 | 0.0065 |
| GO:0036094 | F | small molecule binding | 28 | 17352 | 0.00028 | 0.0065 |
| GO:0004497 | F | monooxygenase activity | 7 | 1728 | 0.00069 | 0.014 |
| GO:0043169 | F | cation binding | 27 | 17543 | 0.00084 | 0.015 |
| GO:0046872 | F | metal ion binding | 26 | 17104 | 0.0013 | 0.022 |
| GO:0016709 | F | oxidoreductase activity, acting on paired donors, with incorporation or reduction of molecular oxygen, NAD(P)H as one donor, and incorporation of one atom of oxygen | 5 | 1008 | 0.0018 | 0.028 |
| GO:0005829 | C | cytosol | 25 | 10311 | 6.70E-07 | 0.00015 |
| GO:0009570 | C | chloroplast stroma | 12 | 2613 | 2.10E-06 | 0.00015 |
| GO:0044435 | C | plastid part | 1 | 5866 | 1.50E-06 | 0.00015 |
| GO:0044434 | C | chloroplast part | 17 | 5295 | 1.70E-06 | 0.00015 |
| GO:0005737 | C | cytoplasm | 60 | 47682 | 1.50E-06 | 0.00015 |
| GO:0044444 | C | cytoplasmic part | 54 | 40994 | 4.90E-06 | 0.00029 |
| GO:0009507 | C | chloroplast | 29 | 15333 | 1.00E-05 | 0.00051 |
| GO:0009532 | C | plastid stroma | 12 | 3152 | 1.40E-05 | 0.00062 |
| GO:0044424 | C | intracellular part | 66 | 60136 | 3.00E-05 | 0.0012 |
| GO:0005622 | C | intracellular | 66 | 60496 | 4.10E-05 | 0.0015 |
| GO:0044446 | C | intracellular organelle part | 34 | 21339 | 4.80E-05 | 0.0016 |
| GO:0044422 | C | organelle part | 34 | 21477 | 5.50E-05 | 0.0016 |
| GO:0009536 | C | plastid | 31 | 19432 | 0.00013 | 0.0036 |
| GO:0043231 | C | intracellular membrane-bounded organelle | 61 | 54543 | 0.00015 | 0.0039 |
| GO:0043227 | C | membrane-bounded organelle | 61 | 54652 | 0.00016 | 0.0039 |
| GO:0043229 | C | intracellular organelle | 61 | 55574 | 0.00032 | 0.0072 |
| GO:0043226 | C | organelle | 61 | 55713 | 0.00036 | 0.0075 |
| GO:0044464 | C | cell part | 67 | 65148 | 0.00045 | 0.0086 |
| GO:0005623 | C | cell | 67 | 65175 | 0.00046 | 0.0086 |
| GO:0009526 | C | plastid envelope | 9 | 2745 | 0.00054 | 0.0094 |
| GO:0044432 | C | endoplasmic reticulum part | 9 | 2755 | 0.00056 | 0.0094 |
| GO:0031976 | C | plastid thylakoid | 8 | 2249 | 0.00067 | 0.0097 |
| GO:0009534 | C | chloroplast thylakoid | 8 | 2227 | 0.00063 | 0.0097 |
| GO:0005783 | C | endoplasmic reticulum | 14 | 6204 | 0.00068 | 0.0097 |
| GO:0042170 | C | plastid membrane | 6 | 1238 | 0.00068 | 0.0097 |
| GO:0048046 | C | apoplast | 8 | 2502 | 0.0013 | 0.018 |
| GO:0042175 | C | nuclear outer membrane-endoplasmic reticulum membrane network | 8 | 2658 | 0.0019 | 0.024 |
| GO:0005789 | C | endoplasmic reticulum membrane | 8 | 2645 | 0.0019 | 0.024 |
| GO:0031969 | C | chloroplast membrane | 5 | 1036 | 0.002 | 0.024 |
| GO:0009579 | C | thylakoid | 8 | 2728 | 0.0023 | 0.027 |
| GO:0031967 | C | organelle envelope | 10 | 4426 | 0.0042 | 0.049 |
