# Supplementary material for: Discovery of miRNAs and Development of Heat-Responsive miRNA-SSR Markers for Characterization of Wheat Germplasm for Terminal Heat Tolerance Breeding
Source: Front Genet. 2021 Jul 28;12:699420. doi: 10.3389/fgene.2021.699420 (PMC8356722; doi:10.3389/fgene.2021.699420)
Supplement: Supplementary file 5 [file Data_Sheet_5.docx]

| **Supplementary Table S5: Summary of physiological/biochemical trait data analysis of genotypes studies during the present study. The table shows contrasting mean difference for all the five traits in heat tolerant and heat susceptible genotypes.** | | | | | | | | | | | | | | | | | | | | | |
| --- | --- | --- | --- | --- | --- | --- | --- | --- | --- | --- | --- | --- | --- | --- | --- | --- | --- | --- | --- | --- | --- |
| **Population/Sub-population "of"** | **Canopy Temperature Depression** | | | | **Relative Water Content** | | | | **Membrane Stability Index** | | | | **SPAD Chlorophyll** | | | | | **Proline** | | | |
|  | **Normal Sown** | | **Late sown** | | **Normal Sown** | | **Late sown** | | **Normal Sown** | | **Late sown** | | **Normal Sown** | | **Late sown** | | | **Normal Sown** | | **Late sown** | |
|  | **10DAA** | **20DAA** | **10DAA** | **20DAA** | **10DAA** | **20DAA** | **10DAA** | **20DAA** | **10DAA** | **20DAA** | **10DAA** | **20DAA** | **10DAA** | **20DAA** | | **10DAA** | **20DAA** | **10DAA** | **20DAA** | **10DAA** | **20DAA** |
| **All genotypes** | 3.23 | 3.35 | 3.35 | 3.03 | 83.35 | 83.41 | 84.17 | 81.06 | 86.30 | 92.62 | 91.91 | 85.16 | 41.91 | 37.95 | | 43.15 | 38.01 | 113.06 | 135.64 | 108.49 | 150.19 |
| **Heat Susceptible Genotypes** | 2.73 | 2.59 | 2.77 | 2.03 | 82.05 | 82.16 | 83.98 | 79.45 | 85.45 | 92.34 | 92.17 | 82.20 | 39.61 | 35.05 | | 42.97 | 35.53 | 94.62 | 109.33 | 96.35 | 121.19 |
| **Heat Tolerant Genotypes** | 3.49 | 3.75 | 3.66 | 3.57 | 84.04 | 84.08 | 84.27 | 81.93 | 86.76 | 92.77 | 91.78 | 86.75 | 43.14 | 39.51 | | 43.25 | 39.35 | 122.99 | 149.80 | 115.02 | 165.81 |
| DAA: Days after anthesis | | | | | | | | | | | | | | | | | | | | | |
